# Penile melanoma: a 20-Year analysis of six patients at the National Cancer Institute of Peru, Lima

**DOI:** 10.3332/ecancer.2017.731

**Published:** 2017-04-04

**Authors:** Andres Guevara Jabiles, Edilberto Yabar Del Mar, Gilmer Arcenio Diaz Perez, Fernando Quiroa Vera, Luis Meza Montoya, Carlos Manuel Morante Deza

**Affiliations:** Urological Oncology Department, National Cancer Institute, Lima 34, Peru

**Keywords:** melanoma, penis, inguinal lymphatic metastasis

## Abstract

Melanoma of the urogenital tract is extremely rare, accounting for less than 0.1% of melanoma cases. The global literature currently describes only 220 cases of penile melanoma, most commonly located on the glans penis.

**Results:**

Penile melanoma accounts for 0.7% of penile cancers and 0.18% of melanoma cases. The average patient age for the six cases we reviewed was 44.5 years. Three of these cases (50%) involved ulceration, one case (16.7%) involved lymphovascular invasion, and three cases (50%) involved mitosis ≥ 1 mm^2^ (0.38–4.7 mm). The average depth of invasion (Breslow) in the five cases that reported this measure was 2.1 mm (0.38–4.7 mm). Applying the American Joint Committee on Cancer tumour, node, metastases (AJCC TNM) staging system, one case was Tx (16.7%), two cases were T1 (33.3%), one case was T2 (16.7%), one case was T3 (16.7%), and one case was T4 (16.7%). Five cases (83.3%) involved wide local resection, and only one case involved partial penile amputation. Four cases (66.7%) involved primary bilateral inguinocrural lymphadenectomy, and only one of those four cases involved lymphatic metastatic disease. One case (16.7%) involved lymphatic recurrence at 12 months in a patient who survived 38 months. One case (16.7%) involved local recurrence at 13 months in a patient who has survived 53 months. Eighty per cent of these patients remain alive, with no evidence of disease after an average follow-up of 96.7 months.

**Conclusion:**

Penile melanoma prognosis depends on the initial clinical stage of the disease. The risk of lymphatic involvement seems to be related to the same risk factors used to assess cutaneous melanoma. Clinicians can thus assess penile melanoma using the AJCC staging system. The use of sentinel lymph node biopsy to determine staging is also becoming more common and could replace prophylactic bilateral inguinal dissection.

## Introduction

Melanoma is a type of skin cancer whose incidence is increasing in both sexes. Melanoma mortality is also high, with an average life expectancy loss of 20.4 years compared to other types of cancer [2]. Clinical subtypes of melanoma are described by where they appear: in skin damaged by sun exposure; skin undamaged by sun exposure; acral or subungual areas; and non-cutaneous, or mucosal melanoma. Non-cutaneous melanoma is less common and appears in mucosa such as the head and neck, ano-rectal area, or gastrointestinal tract, and at sites in the genitourinary tract, such as the vagina, vulva, penis, or urethra. Mucosal and cutaneous melanoma have distinct causes, risk factors, presentation, genetic profiles, responses to treatment, and progression patterns. The prognosis for mucosal melanoma is worse than for cutaneous melanoma, suggesting that patients require more aggressive treatment [3, 4].

**Figure 1.**
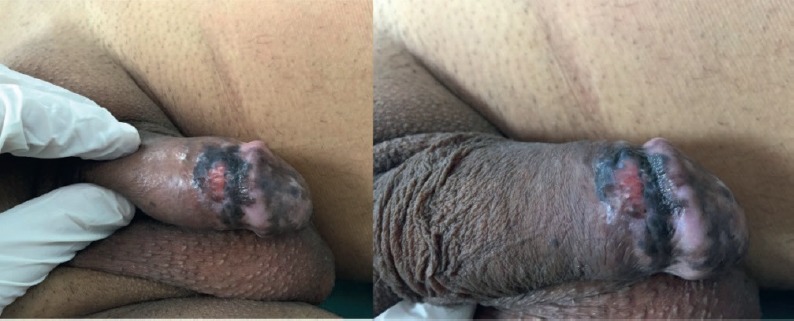
Pigmented, ulcerated lesion on the foreskin and the glans penis.

**Figure 2.**
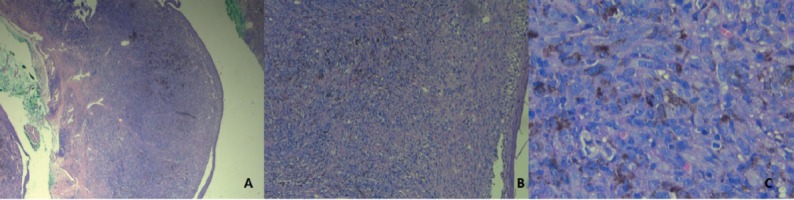
Microscopy of biopsy of infiltrating melanoma. HE-Stain.

Primary penile, urethral, or scrotal melanoma is rare. Muchinson reported the first case in 1859, and the global literature contains only 220 reported cases to date [3]. Penile melanoma accounts for 1.4% of all penile cancers and less than 0.1% of melanoma cases [1, 5]. Penile melanoma results from a malignant transformation of melanocytes in the neuroectodermal layer. Depending on their location on the penis, melanocytes can be considered cutaneous or mucosal. Penile melanomas that appear on penile skin or on the foreskin require examination and treatment following guidelines for cutaneous melanoma. However, penile melanomas appearing in the glans penis, meatum, urethra, crown, or the internal foreskin are considered mucosal [1, 6]. The main challenge in penile melanoma, beyond determining cutaneous or mucosal origin, is early detection. The disease appears as a painless, pigmented lesion that gradually grows larger and then ulcerates. Dermatoscopy is useful for distinguishing penile melanoma from benign lesions such as melanosis, but patients with such lesions require a biopsy (Figures 1 and 2) [7].

Diagnosis of penile cancer requires histopathology showing increased activity of atypical cells and detachment of pigmented dermal cells. Asymmetry, confluence of nest cells, atypical cells, and necrosis of melanocytes are also factors in diagnosis. Immunohistochemistry can be useful for detecting poorly differentiated melanomas. The markers more commonly used are melan-A, HMB 45 and S-100 protein. Serum lactic dehydrogenase should also be measured and the patient should have a full-body computed tomography (CT) or a positron emission tomography (PET) scan, or both to determine staging. Testing for the c-KIT mutation is also useful for deciding whether the patient requires chemotherapy [6, 8].

Most authors describe melanoma of the penis and glans penis using the three-stage system. Stage A includes localised disease in the penis, regardless of the depth of invasion; stage B includes melanoma involving the inguinal lymph nodes; and stage C refers to disseminated metastatic disease. Other authors use the AJCC TNM Staging System [9], which relies on the Breslow depth of invasion, excluding only melanoma of the urethra [3, 10]. Treatment is surgical and includes conservative procedures for localised disease and radical surgeries for locally advanced cases. Advanced cases, however, can also involve surgery to prepare for palliative care and systemic chemotherapy.

The poor prognosis for penile melanoma results from diagnosis at an advanced stage and the aggressiveness of the disease. Early-stage penile melanoma is curable, but the disease is rare. We are therefore reporting the experience of treating and following six cases of penile melanoma over the past 20 years.

## Materials and methods

We conducted a retrospective observational using the database of the National Cancer Institute of Peru (INEN in Spanish) for the period January 1996 to August 2016. This database contained six reports of penile melanoma cases. These six patients came to INEN with melanoma that was either clinically suspected, confirmed via biopsy histology at another institution and/or diagnosed and treated at another institution. Each patient provided a comprehensive medical history, had his inguinal region palpated, and had either a biopsy or a review of pathological slide by an INEN oncology pathologist. Biopsy quality determined the current extent and size of the lesion, depth of invasion in millimetres (Breslow), T stage based on AJCC TNM-staging system [9], ulceration, lymph node invasion, number of mitoses per field, and satellite nodules. Immunohistochemistry did not use any special stain for any of the six cases, and patients did not have sentinel lymph node biopsies. The pathology sample was also used to evaluate resection margin. Two patients had abdominopelvic ultrasound and a chest X-ray to evaluate the extent of their disease and four patients had a CT scan. Patients then came for follow-up every three months, having physical examinations and image examinations to identify any recurrence and recommend appropriate treatment. We also conducted a descriptive analysis of these six cases, using central, median, and average tendencies; percentage values; and tables showing clinical characteristics, disease-free survival time, and overall survival.

## Results

INEN has reported six cases in the past 20 years of male patients diagnosed with penile melanoma that the Institute admitted. These six patients represent 0.7% of all penile cancers and 0.18% of melanomas. Five of these patients had a biopsy at another institution (including three excisional biopsies), and five were referred to INEN with a melanoma diagnosis. Only one case did not involve review of pathological slide, as this patient had received multidisciplinary care that included surgery and chemotherapy. This patient had disease progression when he was admitted to the Institute.

The average patient age was 44.5 years, within a range of 17–60 years, and three patients had been circumcised. Clinical presentation was evidence of a pigmented lesion gradually increasing in size, with ulceration in five cases (83.3%). One patient had a lesion that was not ulcerated. Medical history and physical examination established the site of each lesion: three (50%) on the glans penis (two in the coronal sulcus and one on the glans), and three lesions on the skin (two on the foreskin and one on the skin of the body of the penis). Four of six patients (66.7%) did not have palpable inguinal adenopathy at examination.

Five of the six patients (83.3%) had additional testing for secondary lesions, but results were negative (two had abdominal ultrasound and a chest X-ray and three had a CT scan). One patient (16.7%) did not have additional testing.

**Figure 3.**
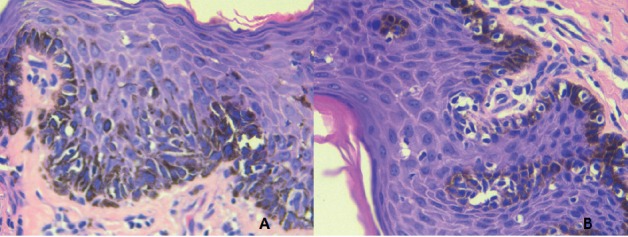
Microscopy of surgical melanoma *in situ* sample. HE-Stain.

Five cases had a pre-surgical biopsy reported as malignant, infiltrating melanoma, and only one (16.7%) was reported as melanoma *in situ* (Figure 3). That result was inconsistent with a previous biopsy, which identified infiltrating melanoma with a Breslow measurement of 1.6 mm. We ruled out the inconsistency as resulting from a regression process, as we found no evidence of histopathological characteristics in our review of the case. The excisional biopsy performed at the other institution likely included the only infiltrating part of the melanoma.

With regard to the pathology results; three (50%) presented ulceration and two (33.3%) absence of ulceration; four (66.7%) did not present lympovascular invasion, one (16.7%) presented lymphovascular invasion and one (16.7%) was unassessable due to the degree of necrosis; three (50%) presented mitosis ≥ 1 mm^2^, two (33.3%)with the absence of mitosis, and one (16.7%) was unassessable; none presented satellite nodules. The average depth of invasion (Breslow) of the five reported cases were 2.1 mm (0.38–4.7) and according to the AJCC/TNM staging system [8]; one (16.7%) was Tx, two (33.3%) were T1, one (16.7%) was T2, one (16.7%) was T3, and one (16.7%) was T4 (Table 1).

Of the six melanoma cases reported, five (83.3%) had wide local resection and one (16.7%) involved partial penile amputation. Sentinel lymph node testing was not carried out in any of the cases. However, four cases (66.7%) involved primary lymphadenectomy (three bilateral inguinocrural and one bilateral ilio-inguinocrural) of which two presented initial palpable adenopathies, both with negative pathological results and two without initial palpable adenopathies, of which only one presented lymph node metastasis.

According to the three-stage system, four (66.6%) are located on the penis and one (16.7%) is located at a regional level. There is no report of case three having depth of invasion or regional lymph node assessment; therefore, it could not be staged. None of the cases started with distant disease.

After surgical treatment, five (83.3%) cases went to observation, while one (16.7%) received chemotherapy with dacarbazine for 10 sessions at another institution. Two (33.3%) of the six cases presented documented recurrence. One case involved local recurrence on three occasions (case 5) with the first recurrence at 13 months. The patient underwent multiple local surgical resections to treat the recurrences. The patient is currently alive with a new local recurrence with a survival rate of 63 months. In another case (case 3), the patient had bilateral regional inguinal lymph node recurrence at 12 months regardless of having received adjuvant chemotherapy with dacarbazine at another institution post-surgery. The patient continued receiving dacarbazine and interferon to treat the regional recurrence. However, the patient passed away because of progression 38 months after diagnosis. One patient was lost sight of post-operative without further follow-up. The average follow-up time for the five patients who kept being monitored was 102 months.

Currently, four (80%) of the patients are alive and only one presents local recurrence. Follow-up time varies from 6–221 months; three with early stage A and one with stage B or regional. The only deceased case has a recorded survival time with the disease of 38 months due to progression of the disease and shows no record of depth invasion since it did not have adequate pathological slide review or pathological lymph node staging, since the patient was diagnosed and treated at another institution (Table 2).

## Discussion

Penile melanoma is a rare pathology that presents itself at an average age of 65 years [1, 3] unlike cutaneous melanomas that appear at 59 years of age [2]. In our series of cases, the average age was 44.5 years, which is less than what the literature reports, probably due to a case of a 17-year-old patient (case 2). For mucosal melanomas, most cases are located on the glans (55%) of which 28% are located on the meatus and 9% on the corona or balanopreputial sulcus; the second most frequent location is the urethra (36%) and finally, the foreskin and frenulum (9.1%) value similar to our series of cases in which 50% of the reported cases were in the glans (2 on the balanopreputial sulcus and 1 on the ventral side of the glans). However, there were no records of urethral melanomas. Of the melanomas located on the skin of the penis, 28% of reported cases are located on the foreskin and 9% on the skin of the body of the penis [8], similar values to the 33.3% and 16.6% in our records. Penile melanoma usually presents itself as a black, blue, or brown pigmented lesion or it can even be amelanotic. Ulceration of the lesion occurs in 39% to 66% of cases [8, 10]; in our series, it goes up to 83.3% of cases.

The data described up to now regarding penile melanoma is still insufficient in determining appropriate management and accurate prognosis. If the melanoma appears on the skin of the penis or on the external foreskin, some of the authors mention that it should be studied and treated following guidelines for cutaneous melanoma. However, if it appears on the glans, meatus, or urethra, corona or internal foreskin, it is considered of mucosal origin and is therefore a more aggressive disease with a poor prognosis [1, 6, 8]. However, van Geel [10] in a 19 case report of mucosal penile melanoma and in 47 cases reviewed in the literature, concludes that regardless of the origin and its location on the penis, the most important prognosis factor is clinical staging of the disease at the time of diagnosis (localised vs. lymph nodal vs. metastatic) also for the cutaneous melanomas; in other words, those originated in the urogenital tract should be assessed by histopathological characteristics already outlined in the current guidelines for cutaneous melanoma to classify the risk of developing recurrence and/or progression. These characteristics include depth of invasion (Breslow), the presence or the absence of ulceration, mitotic index, Clark, vertical or horizontal growth pattern, the presence or the absence of satellite nodules, lymphovascular invasion, size of the tumour and regression pattern [2, 10].

Penile melanomas usually present a poor prognosis compared to cutaneous melanomas. However, this could be explained by the delay in diagnosis due to the difficult visibility of the lesions and because of the high incidence of vertical growth pattern presented in melanomas of the glans and the urethra, similar to what happens with nodular cutaneous melanomas. The literature reports 70% to 100% of these types of growth. However, in our series of cases, only one patient presented this pattern of growth (vertical) in the biopsy, while others did not present this. There are also other factors that have proven to be worse prognosis factors, such as depth of invasion > 3.5 mm, the presence of ulceration, the presence of microsatellite lesions, and size of lesion > 15 mm in diameter [10, 11].

Depth of invasion seems to be the most important risk factor in determining risk of metastasis, as shown by van Geel in the study that proved that patients with a Breslow < 3.5 mm presented better survival and it was Sánchez-Ortiz [1] in the 2005 study that reported that patients with a Breslow ≤ 1.5 mm did not present pathological lymph node disease regardless of the initial clinical adenopathies. In the cases we reviewed, neither of the two cases with Breslow ≤ 1.5 mm presented lymph node disease and just one (16.7%) with a Breslow depth of 3.2 mm (case 1) presented lymph node metastasis. However, another case, (case 5) with greater infiltration (4.7 mm) and palpable adenopathies did not present pathological lymph node disease, which suggests that metastatic lymph node compromise not only depends on the depth of invasion, but also on other histopathological factors.

Mitosis index and the presence of ulceration are also considered risk factors for lymph node disease and at the same time, prognosis factors. In our series, there was only one case (16.7%) with inguinal lymph node compromise from the beginning, regardless of the absence of clinically palpable adenopathies. It is the case of a patient (case 1) who underwent wide local resection at another institution for infiltrating melanoma with a Breslow of 3.2 mm, positive lymphovascular invasion, the presence of ulceration and mitosis index 2 and a month later had broadening of margins with results for melanosis and a bilateral inguinocrural dissection (BICD) with a result of 16/40 positive lymph nodes, none with macro metastasis. The patient has not presented recurrence, with a global survival rate and disease free for 221 months to date, which suggests that patients with minimal lymph node disease or with micrometastasis could be cured with surgical treatment, as reported by Gershenwald in the study about cutaneous melanoma [12], even without pelvic lymphadenectomy.

The most important prognosis factor when it comes to a non-localised disease is the lymph node compromise at the time of diagnosis. 20% of the cases of penile melanoma reported in the literature had pathological compromise [8, 11] and according to van Geel’s study [10], the risk of presenting pathological lymph node disease in patients with palpable adenopathies is between 30% and 40%, unlike those which were not palpable with a risk of only 16%; a value which is repeated in our report. Thus, the importance of why patients with cutaneous melanoma without clinical evidence of lymph node compromise who present risk factors for regional disease should be assessed with sentinel lymph node biopsy, that is why some authors who describe penile melanoma cases currently recommend the use of this procedure with patented blue dye and a tracking radioisotope in order to avoid morbidity caused by bilateral inguinocrural lymphadenectomy. It has been shown that a bilateral inguinocrural dissection (BICD) does not produce greater survival benefits in patients with localised penile disease [3, 13]. Therefore, a sentinel lymph node biopsy should be considered in melanoma cases with risk factors for lymph node compromise, despite its use in penile melanoma, a comparison with prophylactic BICD has not been demonstrated yet with randomized trials due to its rarity [14].

The surgical margin of the excised tissue is another important factor determining the disease-free survival time (DFST), since the only patient who presented a local recurrence was case 5. Despite having a Breslow thickness of 4.7 mm, the presence of ulceration in the excised tissue and mitosis of 3–4 mm^2^, this was the only case involving a compromised surgical margin. Local recurrence was seen on up to three occasions, after the patient underwent repeated local excisions. The high local recurrence rate could be explained by the failure to comply with the necessary standards for free margins of 2 cm in lesions ≥ pT2 in the four local excisions previously carried out [15]. The patient is currently alive and presenting a new local recurrence without regional or metastatic disease after 63 months of monitoring, which could be explained by the prophylatictic bilateral ilio-inginocrural excision to which he was subjected.

Median survival for melanomas of the penis is 28 months, with a global survival rate (SV) after five years of 18–31% [1, 10]; however, survival needs to be better assessed, according to stages of the melanoma and depth of invasion, as demonstrated by the report of cases by B Ruschi and the review by van Geel. Clinical stage A with an invasion of less than 3.5 mm presents a better survival rate of 63–75% at 3 years and 33.3–39% at 5 years [8, 11]. In our study, the median DFST of the five patients is 85 months, while median global survival is 102 months (ranging from 6 to 221 months), with a global survival rate at five years of 66.6%

The treatment proposed for stages located in the glans and the distal urethra is conservative surgery (wide local excision, urethrectomy, glandectomy or partial amputation of the penis) because negative margins suffice, since the more radical approaches have not demonstrated greater benefits [3]. They report a 15–30% local recurrence rate after surgery, mainly in the cases where the depth of invasion has reached the urethral mucosa, with multifocal tumours or inadequate margins that do not meet the standards for cutaneous melanoma [2], as happened in our study, where just one case (16.7%) presented local recurrence, probably owing to histological risk factors and inadequate margins.

Routine bilateral inguinocrural lymphadenectomy is not indicated in stages A, since the percentage of nodal engagement in the early stages is low and depends on the depth of invasion, as reported in the study of Sánchez-Ortiz [1] where only 11% presented inguinal lymph node metastasis, which is similar to the figure in our series of cases, where only one (16.7%) is reported out of six cases. That said, other authors suggest that it could be the only metastatic site, and the inguinocrural lymphadenectomy could therefore have a curative effect. This is the reason why sentinel node biopsy could be used for staging [8] in cases of localised melanoma with a depth of at least 1 mm, Clark IV or V and/or those that present ulceration. Depending on the result of the sentinel node, a bilateral inguinocrural lymphadenectomy could be proposed since it has been demonstrated that patients with minimal nodal metastasis can be cured [3]. With this new recommendation, prophylactic BICDs in stages A should be substituted and only carried out for therapeutic purposes as long as the institute first carries out a sentinel node study. The sentinel node biopsy was not used on any patient owing to the scarcity of cases and because this is retrospective information.

The absence of a pathologic nodal study involving sentinel nodes in the case of infiltrating melanoma could be a factor in poor prognosis, as happened with the sole reported case (case 3), which presented recurrence at the nodal level after 12 months with specific survival of 38 months.

In clinical stages B or C, a routine bilateral ilio-inguinocrural dissection (BIICD) has palliative indication only to reduce local complications owing to the high volume of disease [1, 16]. The prognosis for the non-localised stages is poor, with a reported two-year survival rate of 0% despite multiple treatments [3]; nonetheless, the prognosis in clinical stage B is variable depending on the disease volume. Minimal nodal involvement or micrometastasis present a better prognosis than cases involving macroscopic or clinically palpable disease. Consequently, the role of the bilateral inguinocrural lymphadenectomy could be curative, as also happens in cases of cutaneous melanoma, and as was reported in our series with case 1. Wider nodal disection towards the pelvic region is indicated where there is evidence of palpable macroscopic disease in the inguinal region, that is, in clinical stage B or clinical stage III according to the AJCC, when there is imaging evidence of pelvic metastasis, when more than four inguinal nodes are reported to be involved, with micro metastasis, or where there is involvement of Cloquet’s node [17]. Radiotherapy has not demonstrated any greater benefits and chemotherapy combined with immunotherapy has been used but without being able to estimate the benefits for survival owing to the low number of cases. Since 20% of mucosal melanoma display c-Kit mutations, c-Kit tyrosine kinase inhibitors could be of some use [1, 3], although the molecular data gathered do not specify this information, since this is a retrospective study.

## Conclusions

Melanoma of the penis is a rare disease that presents with the appearance of a pigmented lesion, which is often located on the glans and may be treated with conservative surgery in the majority of cases, depending on the clinical stage and the depth of invasion found at the time of diagnosis. The risk of nodal involvement appears to be related to the same risk factors as in cutaneous melanoma, and therefore, the AJCC staging system may be used. The majority of cases described in the literature describe wide local incisions, with good survival rates over the long term; nonetheless, many reports describe the prognosis for patients with melanoma of the penis as poor, probably because of delays in diagnosis and the presence of suspicious inguinocrural adenopathy indicative of advanced disease. At the moment, there are no adjuvant therapies that improve the survival rate for patients with advanced melanoma of the penis. However, molecular information is useful for the application of systemic therapies. The use of the sentinel node biopsy is considered to be one of the most important prognostic factors for melanoma and should therefore be considered in cases of localised disease of the penis that present risk factors for occult metastasis. Prophylactic BICDs should be avoided owing to their high incidence of morbidity and should only be carried out only where sentinel node biopsies have been positive, at clinical stage B or in cases of bulky inguinal disease.

## Figures and Tables

**Table 1.** Clinical and histopathological characteristics of the six penile melanoma cases at INEN.

Case	Age (y)	Year	Place	Clinic	Clinical inguinal lymph nodes	Breslow (mm)	Ulceration	Mitosis (mm^2^)	LVI
1	40	1998	Penile skin	Pigmented ulceration	Neg	3.2	Present	2	Pos
2	17	2004	Glans	Pigmentation	Pos bilateral	0.38	Absent	Absent	Neg
3	39	1994	Foreskin	Pigmented ulceration	Neg	NA	NA	NA	NA
4	58	2001	Balanopreputial sulcus	Pigmented ulceration	Neg	0.75	Absent	Absent	Neg
5	53	2011	Foreskin	Pigmented ulceration	Pos bilateral	4.7	Present	3 to 4	Neg
6	60	2016	Balanopreputial sulcus	Pigmented ulceration	Neg	1.6	Present	1	Neg

LVI: lymphovascular invasion, NA: not assessable, Neg: negative, Pos: positive, y: years.

**Table 2.** Treatment, pathological staging, and follow-up of the 6 penile melanoma cases at INEN.

Case	Primary treatment	AJCC (2010)	Recurrence	Time (m)	Treatment	Follow-up (m)
1	LWR + BICD	TxN3Mx	No			AWD (221)
2	LWR + BICD	T1aN0M0	NA			NA
3	LWR	TxNxM0	Inguinal bilateral	12	CT (DTIC, IFN)	DRD (38)
4	LWR + BICD	T1aN0M0	No			AWD (221)
5	Circumcision + BIICD	T4bN0M0	Penis (x3)	13	LWR (x3)	AWR (63)
6	Partial penile amputation + BICD	T2bN0M0	No			AWD (6)


AWD: Alive without disease, AWR: Alive with recurrence, BIICD: Bilateral Ilio-inguinocrural dissection, BICD: Bilateral inguinocrural dissection, CT: chemotherapy, DTIC: Dacarbazine, DRD: Disease-related death, IFN: Interferon, LWR: Local wide resection, m: months.
